# Garlic (*Allium sativum* L.): a potential unique therapeutic food rich in organosulfur and flavonoid compounds to fight with COVID-19

**DOI:** 10.1186/s12937-020-00643-8

**Published:** 2020-11-18

**Authors:** Sucheta Khubber, Reza Hashemifesharaki, Mehrdad Mohammadi, Seyed Mohammad Taghi Gharibzahedi

**Affiliations:** 1 Center of Innovative and Applied Bioprocessing, Mohali, 140306 India; 2 CCS Haryana Agricultural University, Hisar, 125004 India; 3 Department of Research and Development, Nova Harmony LLC, Santa Monica, California USA; 4 Department of Food Technology Research, National Nutrition and Food Technology Research Institute, Faculty of Nutrition Sciences and Food Technology, Shahid Beheshti University of Medical Sciences, Tehran, Iran; 5 Young Researchers and Elite Club, Lahijan Branch, Islamic Azad University, Lahijan, Iran

**Keywords:** COVID-19, SARS-CoV-2, Garlic, Allicin, Quercetin, Antiviral

## Abstract

Coronavirus disease 2019 (COVID-19) is the current major health crisis in the world. A successful strategy to combat the COVID-19 pandemic is the improvement of nutritional pattern. Garlic is one of the most efficient natural antibiotics against the wide spectrum of viruses and bacteria. Organosulfur (e.g., allicin and alliin) and flavonoid (e.g., quercetin) compounds are responsible for immunomodulatory effects of this healthy spice. The viral replication process is accelerated with the main structural protease of SARS-CoV-2. The formation of hydrogen bonds between this serine-type protease and garlic bioactives in the active site regions inhibits the COVID-19 outbreak. The daily dietary intake of garlic and its derived-products as an adjuvant therapy may improve side effects and toxicity of the main therapeutic drugs with reducing the used dose.

Dear Editor,

The most important global health concern is the viral pneumonia outbreak of coronavirus disease 2019 (COVID-19). Over the past few months, the COVID-19 related social distancing seriously affected the psychological, economic, and social well-being features. The COVID-19 epidemic caused by severe acute respiratory syndrome coronavirus 2 (SARS-CoV-2) primarily induces pro-inflammatory cytokines (e.g., IL-1 and IL- 6) and lung inflammation. This virus can also damage vital organs of the host body through the expression of the angiotensin-converting enzyme 2 (ACE2) receptor. Besides, the imbalance between the renin-angiotensin system and ACE2/angiotensin-(1–7)/MAS axis after the SARS-CoV-2 infection enhances comorbidities and multi-organ injuries [1]. So far no effective drugs and vaccines to treat patients with COVID-19 have been reported. Until then, the drug treatments will include remdesivir, umifenovir, favipiravir, lopinavir/ritonavir, ribavirin, hydroxychloroquine, etc. Besides, dietary therapy and herbal medicine as an adjuvant therapy may be one of the efficient strategies to fight against COVID-19. Bioactive constitutes involved in immunomodulatory, antioxidant, and antimicrobial activities in certain foods and herbs may be able to pre-and post-exposure prophylaxis by increasing the activity and number of cytokine suppressors, lymphocytes, natural killer cells, and macrophages. Therefore, herbal product decrease the adverse impacts of antivirals by reducing the used dose and synergically improves the remedy and outcomes by decreasing the inflammation and respiratory symptoms [2].

The presence of sulfur-containing phytochemicals in garlic (*Allium sativum* L.) provides substantial immunomodulatory, anti-inflammatory, anticancer, antitumor, antidiabetic, anti-atherosclerotic, and cardioprotective features. The most important of sulfur constitutes (~ 82%) of garlic thiosulfinates (allicin), S-allyl cysteine sulfoxide (alliin), ajoenes (E- and Z-ajoene), vinyldithiins (2-vinyl-(4H) -1,3-dithiin, 3-vinyl-(4H)-1,2-dithiin), and diallyl (di and tri) sulfide. Also, there are some alliin-derived organosulfur compounds (OSCs) in garlic such as S-allyl-cysteine, S-ally-mercapto cysteine, and N-acetylcysteine [3]. The antiviral potential of garlic against a number of viruses like influenza B, HIV (type 1), vesicular stomatitis virus, herpes simplex virus (types 1 and 2), coxsackievirus species, and gammaretrovirus was earlier demonstrated [4]. Recently, researchers have realized the structure of the main protease of SARS-CoV-2, a serine-type M^pro^ (chymotrypsin-like protease (3CL^pro^)) protease with the kind of amino acids (such as Thr24, Thr26, and Asn119) present in the active site regions (e.g., 6 LU7 and 2GTB). M^pro^ has a considerable structural similarity (~ 96.0%) between types 1 and 2 of SARS-CoV. Since this protease is responsible for the viral replication and the production functional protein as a result of the proteolytic maturation of SARS-CoV-2, the infection rate may be substantially reduced by hindering the cleavage of the viral polyprotein [5]. In an in silico approach on the inhibitory effect of garlic against SARS-CoV-2, seven OSCs of alliin, S-(allyl/ methyl/ethyl/propyl)-cysteine, S-propyl L-cysteine, and S-ally-mercapto-cysteine were considered as possible constitutes to inhibit the M^pro^ of SARS-CoV-2 through H-bonds with this protease. Molecular docking analysis showed that alliin among other OSCs has higher anti-viral potential to prevent COVID-19. This bioactive component alone or in combination with the main therapeutic drug would be an efficient therapy to eradicate SARS-CoV-2 with the lowest side effects and toxicity [6, 7]. Similar findings on the inhibitory effect of phytochemicals extracted from black cumin, black pepper, and ginger was obtained [7]. The concentration of 0.1 mL of garlic clove extract revealed a potent in vivo inhibitory effect against SARS-CoV-1 multiplication, probably due to the formation blocking of structural proteins and genetic materials [8]. The quercetin could also inhibit protease present in SARS-CoV-1 during the multiplication in host cells via blocking the viral attachment stage [9].

Decreasing the rate of viral infection caused by SARS-CoV-2 may be contributed to the presence of organosulfur (e.g., allicin) and flavonoid (e.g., quercetin) compounds in aqueous extracts and essential oils of garlic and their interaction with the M^pro^ protease. The encapsulation of these bioactive substances at the micro- and nano-size drug particles maintains their oxidative stability and bio-functionality and provides their controlled release and delivery to the targeted sites. Finally, the consumption of functional foods prepared by encapsulated/free bioactive compounds of garlic may have a key role in the incidence reduction of COVID-19 in different communities.

## Data Availability

Not applicable.

## Abbreviations

ACE2: Angiotensin-converting enzyme 2
COVID-19: Coronavirus disease 2019
OSCs: Organosulfur compounds
SARS-CoV-2: Severe acute respiratory syndrome coronavirus 2
